# Twiddler’s syndrome in extravascular implantable cardioverter-defibrillator: a case report

**DOI:** 10.1093/ehjcr/ytag186

**Published:** 2026-03-14

**Authors:** Abbie Measom, Robin Collard, Rajesh Chelliah, Harshil Dhutia, Shirley Sze

**Affiliations:** Department of Cardiology, Glenfield Hospital, University Hospitals of Leicester NHS Trust, Groby Road, Leicester LE3 9QP, UK; Department of Cardiology, Glenfield Hospital, University Hospitals of Leicester NHS Trust, Groby Road, Leicester LE3 9QP, UK; Department of Cardiology, Glenfield Hospital, University Hospitals of Leicester NHS Trust, Groby Road, Leicester LE3 9QP, UK; Department of Cardiology, Glenfield Hospital, University Hospitals of Leicester NHS Trust, Groby Road, Leicester LE3 9QP, UK; Department of Cardiology, Glenfield Hospital, University Hospitals of Leicester NHS Trust, Groby Road, Leicester LE3 9QP, UK; NIHR Biomedical Research Centre and Department of Cardiovascular Sciences, University of Leicester, Glenfield Hospital, Groby Road, Leicester LE3 9QP, UK; Department of Population Health Sciences, University of Leicester, University Road, Leicester LE1 7RH, UK

**Keywords:** EV-ICD, Twiddler’s syndrome, Case report, Inappropriate shock

## Abstract

**Background:**

Twiddler’s syndrome is caused by rotation of the pulse generator, which could lead to device malfunction. It has been reported in patients with transvenous and subcutaneous defibrillators but not in those with extravascular implantable cardioverter-defibrillators (EV-ICDs).

**Case summary:**

A 57-year-old man with an extraction for infected transvenous ICD (TV-ICD) underwent an EV-ICD for primary prevention. Device checks pre-discharge and at 6 weeks were unremarkable. However, 3 months post-implant, the patient experienced an inappropriate shock due to oversensing of noise. Chest X-ray revealed lead displacement, with the device rotated nearly 180° with the lead wrapped around it. Surgical revision confirmed lead coiling and torn fascia sutures, consistent with Twiddler’s syndrome.

**Discussion:**

This is the first reported case of Twiddler’s syndrome in a patient with an EV-ICD, presenting with inappropriate shock due to oversensing of noise. Clinicians should consider Twiddler’s syndrome as a potential cause of lead dislodgment and EV-ICD malfunction. Early recognition is crucial to enable timely intervention, restore device function, and prevent further complications.

Learning points

- Twiddler’s syndrome, although rare, should be considered as a potential cause of lead displacement and device malfunction in patients with EV-ICDs.
- Prompt device interrogation and chest X-ray are essential to prevent inappropriate shocks or failure to deliver life-saving therapies in times of ventricular arrhythmias.
- Patient education, secure generator fixation, and an appropriately sized pocket could help prevent Twiddler’s syndrome.

## Introduction

Implantable cardioverter-defibrillators (ICDs) reduce the risk of sudden cardiac death in patients with ventricular arrhythmias.^1^ Transvenous ICDs (TV-ICD) are commonly used but are associated with complications such as vascular injury, cardiac perforation, pneumothorax, and haemothorax. The subcutaneous ICD (S-ICD) was developed to avoid these risks.^2^ Although the S-ICD is effective in preventing sudden arrhythmic death, it does not provide anti-tachycardia pacing and offers limited bradycardia support, typically restricted to the immediate post-shock period.^3^ Due to extra-thoracic lead placement, S-ICD requires higher current for both pacing and defibrillation. This may lead to extracardiac stimulation and necessitates a larger pulse generator.^4^

A newer device, the extravascular ICD (EV-ICD), uses a sub-sternal lead to deliver pacing and defibrillation without entering the vasculature. The Epsila lead is tunnelled beneath the sternum, with the generator placed along the mid-axillary line. Early data suggest safe implantation and effective arrhythmia detection and termination, although long-term outcomes remain unclear.^5^

Twiddler’s syndrome, caused by device rotation, could result in lead displacement and device malfunction.^6^ It has been well described in transvenous pacing systems and SICD, but not in EV-ICD. We report the first case of Twiddler’s syndrome in a patient with an EV-ICD, presenting with inappropriate shock 3 months post-implantation.

## Summary figure

**Figure:**
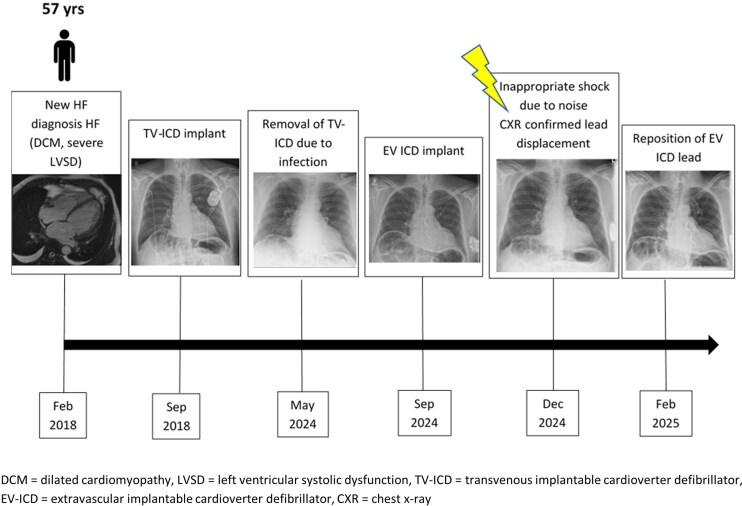
Timeline of events.

## Case presentation

A 57-year-old male presented with breathlessness and peripheral oedema. Cardiac MRI revealed dilated non-ischaemic cardiomyopathy and severe left ventricular (LV) impairment (ejection fraction = 25%) with mild basal anterior, septal, and inferior hinge point late gadolinium enhancement. He was initiated on guideline-directed medical therapy. Six months later, he presented to hospital with palpitations and dizziness. Twenty-four-hour Holter revealed episodes of non-sustained ventricular tachycardia (NSVT). In view of history of cardiomyopathy with persistent severe LV systolic impairment and high arrhythmic risk, a single-chamber TV-ICD was implanted for primary prevention (Summary figure).

In May 2024, the patient reported discomfort and swelling at the TV-ICD site. Examination revealed lateral migration of the device associated with protuberance and tenderness. He was admitted for device reburial; however, intraoperative findings raised concerns for pocket infection and lead displacement, prompting complete system extraction. A repeat echocardiogram demonstrated persistent significant LV systolic dysfunction (LVEF 35%–40%) and device interrogation detected recurrent episodes of NSVT; therefore, decision was made to re-implant an ICD after extraction. After 1 week of antibiotics, device re-implantation options were discussed, including a TV-ICD on the right side or EV-ICD. The patient elected for EV-ICD to reduce infection risk. A chest CT showed no contraindicating anatomy, and an EV-ICD was implanted successfully. Initial device testing showed a satisfactory defibrillator safety margin (DSM) at 40 J. Undersensing of ventricular ectopics (VEs) was resolved by switching the sensing vector from Ring1-Ring 2 (standard set up) to Ring1-Can. Six-week post-implant checks were normal.

**Figure 1:**
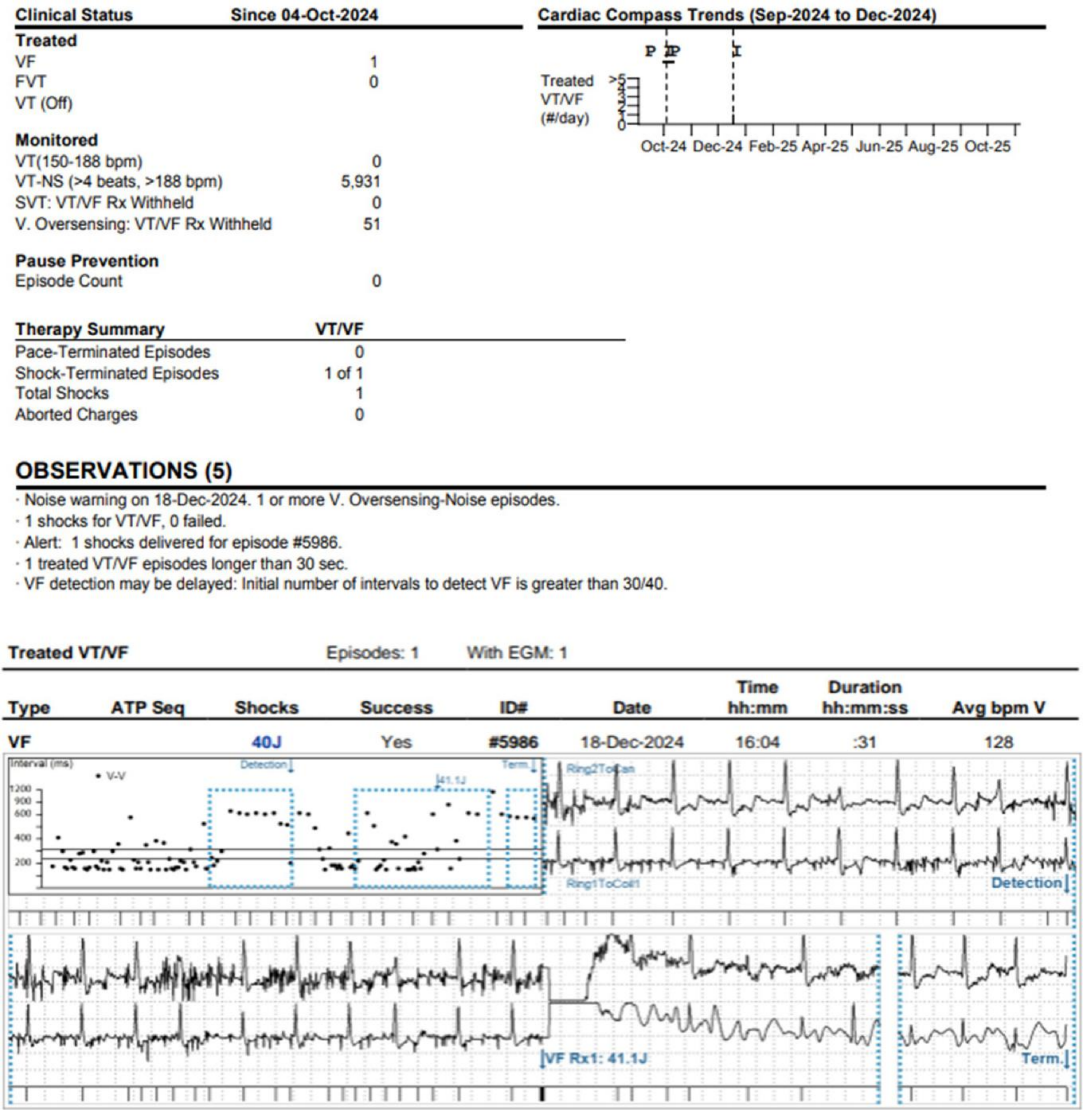
Device interrogation after the patient had a shock. (Top) Extravascular implantable cardioverter-defibrillator device interrogation summary: the device delivered one shock for ventricular fibrillation which was inappropriate due to oversensing of noise episodes. Overall, there were 5931 episodes of non-sustained ventricular tachycardia inappropriately detected due to oversensing noise episodes. (Bottom) Electrogram: 1 × 40 J shock delivered inappropriately for ventricular fibrillation due to oversensing noise episodes.

**Figure 2:**
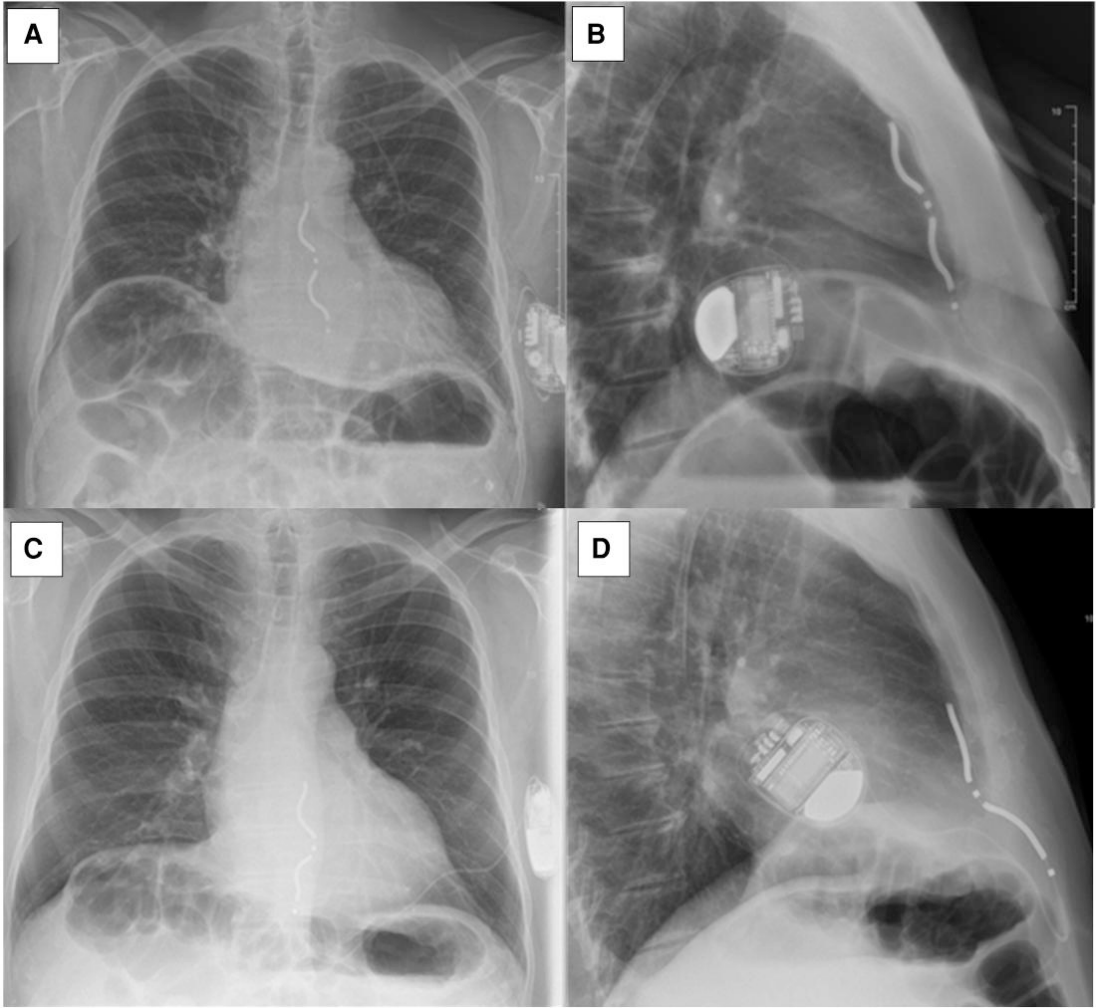
Posterior-anterior and lateral chest X-rays of extravascular implantable cardioverter-defibrillator. (*A*) Posterior-anterior and (*B*) lateral chest X-rays immediately post-implant. (*C*) Posterior-anterior and (*D*) lateral chest X-rays 3 months post demonstrating lead displacement and rotation of device of nearly 180 degrees with the lead coiled around it.

In December 2024, 3 months post EV-ICD implant, the patient received an inappropriate shock (*Figure 1*). Device interrogation revealed noise on two sensing vectors (Ring 1-Can and Ring2-Can); Ring1-Ring2 had a small R wave with undersensed VEs as seen in the post-implant check. The ICD therapies were deactivated to avoid further inappropriate shocks. Chest X-rays showed lead retraction and nearly 180-degree rotation of the generator, with the lead coiled around it (*Figure 2*). The patient denied any trauma or device manipulation. Twiddler’s syndrome was suspected.

**Figure 3:**
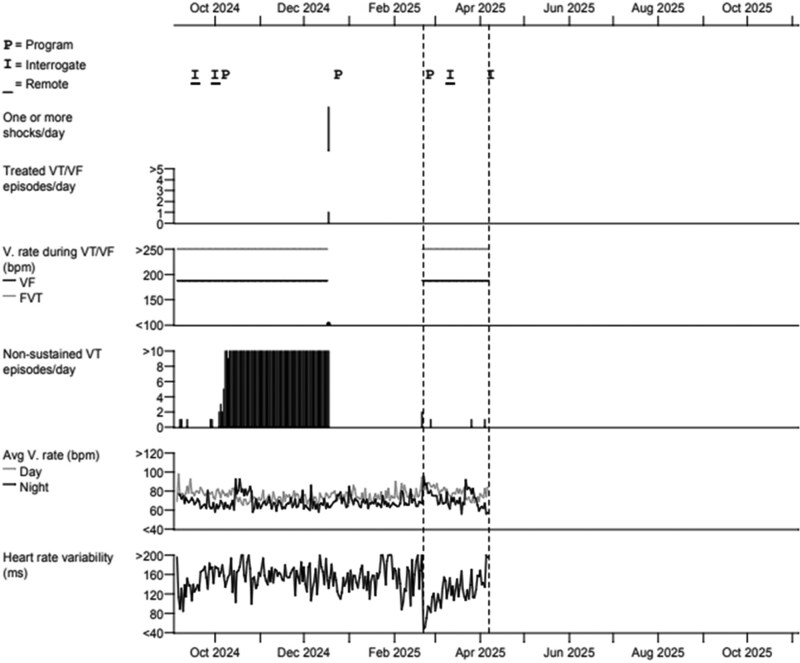
After repositioning of extravascular implantable cardioverter-defibrillator lead (February 2025), there were no further detection of noise episodes, minimal genuine non-sustained ventricular tachycardia, and no further shock therapy in subsequent pacing checks.

The patient underwent lead revision. Intraoperatively, it was found that all three rectus sheath sutures were intact; however, the two generator fixation sutures had torn from the fascia. The old ICD lead was removed, and a new lead was deployed in the sub-sternal space with satisfactory R wave. Defibrillator safety margin testing was successful at 30 J, and post-implant checks were satisfactory. The patient was discharged home following targeted education about device care, including explicit advice to avoid twisting or manipulating the generator and to report any new discomfort, swelling, or unusual sensations at the device site, which may indicate device or lead-related complications requiring prompt evaluation. He continues to do well on follow-up (*Figure 3*).

## Discussion

Twiddler’s syndrome is a rare cause of cardiac device malfunction, with an incidence of 0.1%–2.7%,^7^ due to intentional or unintentional manipulation or rotation of the pulse generator. While Twiddler’s syndrome has been reported previously in patients with transvenous device and S-ICDs,^7,8^ this is, to our knowledge, the first reported case involving an EV-ICD, resulting in an inappropriate shock.

This case highlights the importance of considering Twiddler’s syndrome as one of the causes of lead dislodgement or device malfunction in patients with EV-ICD. Twiddler’s syndrome could result in failure to sense and/or pace, inappropriate shocks, or loss of defibrillation function, all of which pose significant clinical risks.^8^ It could also cause phrenic nerve stimulation with diaphragmatic contraction or pectoral muscle or brachial plexus stimulation resulting in rhythmic arm twitching.^9^ Risk factors include older age, female sex, obesity (due to loose subcutaneous tissue allowing device to rotate in its pocket), psychiatric illness, and a relatively large device pocket.^10^

Early recognition is critical to avoid complications. Diagnosis typically involves electrocardiogram, chest X-ray, and device interrogation.^11^ As described in our case, remote device monitoring post-implant could aid in early detection of device malfunction by flagging abnormal sensing or arrhythmic events. Chest X-ray could confirm lead dislodgment or coiling and device rotation. In most cases of Twiddler’s syndrome, lead replacement or reposition, as well as pulse generator relocation, would be required to restore device function. Preventive measures such as patient education against generator manipulation, creating a tighter device pocket, securing the pulse generator to the fascia, more frequent device interrogation in high-risk populations, have been proposed to reduce the risk of developing this syndrome. Generator position and pocket depth may also influence susceptibility to Twiddler’s syndrome. Although deeper submuscular placement, such as that used for some S-ICD systems, might theoretically improve device stability, this has not been systematically studied in EV-ICD implants and remains an area for future evaluation.

## Conclusion

Twiddler’s syndrome is an important differential diagnosis in patients presenting with cardiac device malfunction including EV-ICD. Diagnosis of Twiddler’s syndrome requires a high level of suspicion due to the variety of presentations. Once diagnosis is confirmed, appropriate treatment including lead revision as well as preventative measures should be initiated as soon as possible to prevent further complications.

## Data Availability

The data underlying this article are available in the article and in its online supplementary material.

## Lead author biography

Dr Shirley Sze is a Cardiology registrar currently based at Glenfield Hospital and an Honorary Academic Clinical Lecturer in Cardiology at the University of Leicester. Her main interests are in heart failure, cardiac devices, arrhythmias, comorbidities, and cardiogeriatrics. She has received funding from the British Heart Foundation to develop patient-centred intervention to improve clinical outcomes of older people living with cardiovascular diseases. She is also part of the European Society of Cardiology Heart Failure Association Scientific Committee on comorbidities.

## Notes

**Consent:** The patient has provided written consent using the *European Heart Journal – Case Reports* Patient Consent form.

## References

[1] Bardy GH, Lee KL, Mark DB, Poole JE, Packer DL, Boineau R, et al Amiodarone or an implantable cardioverter-defibrillator for congestive heart failure. N Engl J Med 2005;352:225–237. 15659722 10.1056/NEJMoa043399

[2] Nso N, Nassar M, Lakhdar S, Enoru S, Guzman L, Rizzo V, et al Comparative assessment of transvenous versus subcutaneous implantable cardioverter-defibrillator therapy outcomes: an updated systematic review and meta-analysis. Int J Cardiol 2022;349:62–78. 34801615 10.1016/j.ijcard.2021.11.029

[3] Knops RE, van der Stuijt W, Delnoy PPHM, Boersma LVA, Kuschyk J, El-Chami MF, et al Efficacy and safety of appropriate shocks and antitachycardia pacing in transvenous and subcutaneous implantable defibrillators: analysis of all appropriate therapy in the PRAETORIAN trial. Circulation 2022;145:321–329. 34779221 10.1161/CIRCULATIONAHA.121.057816

[4] Boston Scientific . *BSX user’s manual: EMBLEM S-ICD and EMBLEM MRI S-ICD*. https://www.bostonscientific.com/content/dam/bostonscientific/Rhythm%20Management/portfolio-group/EMBLEM_S-ICD/Download_Center/359481-001%20EMBLEM%20S-ICD%20PTM_English.pdf (11 October 2025).

[5] Friedman P, Murgatroyd F, Boersma LVA, Manlucu J, O’Donnell D, Knight BP, et al Efficacy and safety of an extravascular implantable cardioverter-defibrillator. N Engl J Med 2022;387:1292–1302. 36036522 10.1056/NEJMoa2206485

[6] Bayliss CE, Beanlands DS, Baird RJ. The pacemaker-twiddler's syndrome: a new complication of implantable transvenous pacemakers. Can Med Assoc J 1968;99:371–373. 4952398 PMC1924435

[7] Jin C, Iwai S, Jacobson J, Ferrick A. A case of twiddler's syndrome with a subcutaneous implantable cardioverter-defibrillator. HeartRhythm Case Rep 2022;8:596–597. 35996701 10.1016/j.hrcr.2022.06.004 PMC9391393

[8] Jabri A, Laiq Z, Nabeel Y. Twiddler's syndrome: an unusual cause of repeated shocks by implantable cardioverter-defibrillator in an asymptomatic patient. Heart Views 2019;20:118–121. 31620258 10.4103/HEARTVIEWS.HEARTVIEWS_45_19 PMC6791089

[9] Nicholson WJ, Tuohy KA, Tilkemeier P. Twiddler's syndrome. N Engl J Med 2003;348:1726–1727. 12711756 10.1056/NEJM200304243481722

[10] Mandal S, Pande A, Kahali D. A rare case of very early pacemaker twiddler's syndrome. Heart Views 2012;13:114–115. 23181182 10.4103/1995-705X.102157 PMC3503355

[11] Tahirovic E, Haxhibeqiri-Karabdic I. Twiddler's syndrome: case report and literature review. Heart Views 2018;19:27–31. 29876029 10.4103/HEARTVIEWS.HEARTVIEWS_89_17 PMC5965012

